# Prospecting sugarcane resistance to Sugarcane yellow leaf virus by genome-wide association

**DOI:** 10.1007/s00122-014-2334-7

**Published:** 2014-06-12

**Authors:** S. Debibakas, S. Rocher, O. Garsmeur, L. Toubi, D. Roques, A. D’Hont, J.-Y. Hoarau, J. H. Daugrois

**Affiliations:** 1 Université des Antilles et de la Guyane, 97157 Pointe-à-Pitre, Guadeloupe France; 2 CIRAD, UMR BGPI, 97170 Petit Bourg, Guadeloupe France; 3 CIRAD, UMR AGAP, 34398 Montpellier, France; 4 CIRAD, UMR AGAP, 97170 Petit Bourg, Guadeloupe France

## Abstract

*****Key message***:**

**Using GWAS approaches, we detected independent resistant markers in sugarcane towards a vectored virus disease. Based on comparative genomics, several candidate genes potentially involved in virus/aphid/plant interactions were pinpointed.**

**Abstract:**

Yellow leaf of sugarcane is an emerging viral disease whose causal agent is a Polerovirus, the Sugarcane yellow leaf virus (SCYLV) transmitted by aphids. To identify quantitative trait loci controlling resistance to yellow leaf which are of direct relevance for breeding, we undertook a genome-wide association study (GWAS) on a sugarcane cultivar panel (*n* = 189) representative of current breeding germplasm. This panel was fingerprinted with 3,949 polymorphic markers (DArT and AFLP). The panel was phenotyped for SCYLV infection in leaves and stalks in two trials for two crop cycles, under natural disease pressure prevalent in Guadeloupe. Mixed linear models including co-factors representing population structure fixed effects and pairwise kinship random effects provided an efficient control of the risk of inflated type-I error at a genome-wide level. Six independent markers were significantly detected in association with SCYLV resistance phenotype. These markers explained individually between 9 and 14 % of the disease variation of the cultivar panel. Their frequency in the panel was relatively low (8–20 %). Among them, two markers were detected repeatedly across the GWAS exercises based on the different disease resistance parameters. These two markers could be blasted on *Sorghum bicolor* genome and candidate genes potentially involved in plant–aphid or plant–virus interactions were localized in the vicinity of sorghum homologs of sugarcane markers. Our results illustrate the potential of GWAS approaches to prospect among sugarcane germplasm for accessions likely bearing resistance alleles of significant effect useful in breeding programs.

**Electronic supplementary material:**

The online version of this article (doi:10.1007/s00122-014-2334-7) contains supplementary material, which is available to authorized users.

## Introduction

Yellow leaf disease of sugarcane (*Saccharum* spp.) is an important and widely spread disease causing severe yield losses, ranging from 20 to 40 % in susceptible cultivars (Lockhart and Cronjé 2000; Zhu et al. 2011; Rassaby et al. 2003; Vega et al. 1997). This disease has been well described (Schenck 2001; Rott et al. 2008) and is caused by the Sugarcane yellow leaf virus (SCYLV), a polerovirus (D’Arcy and Domier 2005) vectored in a persistent, circulative, non-propagative manner by different aphids species as *Ceratovacuna lanigera, Melanaphis sacchari, Rhopalosiphum maidis*, and *R. rufiabdominalis* (Schenck and Lehrer 2000; Zhou et al. 2006). *M. sacchari* has been shown to be the most efficient aphid vector of SCYLV when compared with *R. maidis*, and *R. rufiabdominalis* (Schenck and Lehrer 2000).

Yellow leaf spread by aphids depends on cultivar susceptibility and local epidemiological conditions as well as climatic parameters (Daugrois et al. 2011) and aphid predator populations. The disease is also spread by the use of infected cuttings as seed cane in planting operations. Currently, little is known on the dynamics of the virus within infected sugarcane (circulation and maintenance) with respect to the complex anatomy stalks which emerge from partially persistent root systems across successive crop cycles. In addition, because of asymptomatic and non-specific foliar symptoms (yellowing of leaf midrib), the spread of the disease may be insidious. The only reliable and rapid methods to confirm the presences of the virus are by immunoassay (Schenck et al. 1997) or PCR approaches (Comstock et al. 1998).

SCYLV was first identified in Guadeloupe in 1996 (Daugrois et al. 1999) where the virus is vectored by the aphid *M. sacchari* (Zehntner). This virus is present in all commercial sugarcane cultivars and in all sugarcane growing areas in Guadeloupe, where field SCYLV incidences varied from 0 to 21 % in 2005 depending on cultivars and locations (Edon-Jock et al. 2007). Incidences have since increased to reach, in 2010, between 1 and 72 % for the most susceptible cultivars (personal communication, J.H. Daugrois).

Varietal resistance improvement is the most efficient strategy to control sugarcane diseases, but the lack of knowledge of the genetic basis of yellow leaf resistance makes breeding progress for this disease difficult. Some studies have dealt with the exploration of genotypic variability of resistance to SCYLV in sugarcane germplasm. Indexation of germplasm collections of sugarcane and related species for the presence of the virus revealed potential sources of resistance to yellow leaf disease. Commercial cultivars (*Saccharum* spp.), *S. officinarum*, *S. robustum* and *S. sinense* presented high virus incidence, whereas virus incidence was low for *S. barberi and* *S. spontaneum*, and the related genus *Miscanthus* spp. and *Erianthus* spp. (Comstock et al. 2001; Komor 2011). Until today, only one genetic study was attempted to characterize resistance to yellow leaf disease. Using a QTL approach based on a biparental progeny between a susceptible cultivar and a resistant clone, Costet et al. (2012) tagged the first major quantitative trait allele (QTA) of resistance to SCLYV (named *Ryl*1). Identifying additional QTLs for resistance would allow for greater flexibility in breeding programs, as well as allowing resistance pyramid strategies to be employed. To this end, a survey of the current sugarcane germplasm in a genome-wide association mapping represents an attractive approach to search for additional resistance genes.

The possibility of applying linkage disequilibrium (LD) based studies, or association mapping studies, to identify marker-trait associations in sugarcane has been highlighted earlier given the relatively large extent of LD existing among modern cultivars (Jannoo et al. 1999). The large extent of LD is attributed to the recent breeding history characterized by only few generations separating modern cultivars from a limited number of founder clones. LD is strong in the first five centimorgans and generally drops sharply when markers are 5 cM or more apart, although instances of LD extending up to 10 or 20 cM are not rare (Raboin et al. 2008). Modern sugarcane cultivars (*Sacccharum* spp.) derive from introgressions into the highly polyploid domesticated sugar-producing species *S. officinarum*, (*x* = 10 and 2*n* = 8*x* = 80) of the wild *S. spontaneum* species characterized by different cytotype levels (*x* = 8 and 2*n* = 5*x* − 16*x* = 40 − 128) (D’Hont et al. 1998; Sreenivasan et al. 1987). As a result, sugarcane cultivars have a large and complex genome of about 120 chromosomes (10 Gb) corresponding to about 12 homologous sets of a monoploid genome of 1,500 cM. Notwithstanding genome complexity, several studies have applied association mapping to sugarcane. Using 1,209 polymorphic markers (AFLP and SSR), Wei et al. (2006) detected associations with resistance to four diseases in a collection of 154 cultivars and later on they identified DArT markers related to cane yield and sugar content traits on a sample of 480 genotypes (Wei et al. 2010). These studies confirmed the potential of genome-wide association mapping in the polyploid context of sugarcane.

The purpose of this work was to prospect for sugarcane resistances to SCYLV disease using a genome-wide association approach to identify markers linked to genomic regions involved in the control of the disease.

## Materials and methods

### Plant material

A panel of 189 sugarcane accessions originated from 26 sugarcane breeding programs around the world (supplementary material 1) was planted in two successive trials (A and B) at Station de Roujol, CIRAD, Petit Bourg, Guadeloupe, French West Indies. This panel was composed of a large majority of advanced interspecific sugar-producing commercial hybrids and a very few early-generation interspecific hybrids. Both trials were planted in a randomized complete block design with three complete blocks and a 4 m sugarcane row as an experimental plot. Plots were separated by 1.5 m between line and rows. Trials were conducted during three successive crop cycles. Both trials were planted using cuttings from the germplasm collection of CIRAD Petit Bourg which had been maintained locally for at least 5 years under natural infection. Trial A was planted on 28 September, 2005. Dates of successive crop harvest were 22 May 2006, 26 March 2007 and 17 March, 2008 for plant cane (PC), first ratoon (R1) and second ratoon (R2) crops. Trial B was planted on 26 September, 2007. Dates of successive crop harvest were 11 June 2008, 28 May 2009 and 15 March 2010 for PC, R1 and R2 crop. Due to the inter-annual variation in weather and crop conditions, trials A and B underwent different vector pressures. Population of aphids was abundant in plant cane in trial A, as evidenced by sooty mold that accompanies severe infestations of the aphid in sugarcane (Hall and Bennett 1994) and absence of other sap feeding insects, whereas aphids were sparsely observed in ratoon crops of trial A and in all crop cycles of trial B where no sooty mold was observed.

### Sampling for SCYLV diagnoses by tissue blot immunoassay (TBIA)

In both trials (A and B), assessments of the cultivar panel for yellow leaf were achieved using diagnoses of leaf and stalk infection using tissue blot immunoassay (TBIA). A total of 10–15 first visible dewlap (FVD) leaves were sampled per experimental plot on 5-month-old plants in PC and R2 crop years. Leaf imprints were made by printing a cross section of the bottom part of the midrib of the FVD leaf. In addition, six (PC trial A) and ten (PC trial B and R2 of both trials) stalks were randomly sampled per plot before harvest date. A longitudinal core of 1 cm diameter was taken from an internode of the one-third lower part of the stalk. A stalk imprint was done with a transversal section of the core as done for ratoon stunting disease diagnosis (Davis et al. 1994).

Leaf and stalk imprints were made by applying hand-pressure to the transversal section of plant tissues on a nitrocellulose membrane (Millipore, Protran BA85, 0, 45 µm) for one second.

### SCYLV serological diagnosis of tissue imprints

SCYLV in leaf and stalk samples was diagnosed by following the method described by Schenck et al. (1997) modified by Daugrois et al. (2011). Briefly after being blocked with BSA, the membranes were incubated in anti-SCYLV AS-R2 IgG (kindly provided by Pr B.E.L. Lockhart). The membranes were washed in TBS-Tween (0.05 %), and incubated in anti-rabbit IgG alkaline phosphatase conjugate antibody produced in goat (Sigma-Aldrich A3812). After washing, the membranes were incubated in a substrate solution NBT/BCIP tablets (Sigma-Aldrich B5655). Positive samples and infection intensity of imprints were read on wetted processed membranes under a diascopy binocular microscope.

### Disease resistance traits

For each experimental plot, we estimated the virus incidence in leaves and stalks based on the percentage of positive leaves and stalks, respectively. We also estimated the virus population density within both plant tissues by computing for each plot the means of the infection intensity observed on imprints on the basis of a 0–5 scale. For leaf imprints, this intensity scale was: 0: no vascular bundle infected; 1: infected vascular bundles (ivb) <10 %; 2: 10 % ≤ ivb <50 %; 3: 50 % ≤ ivb <100 %; 4: 100 % ivb with slight coloration; and 5: 100 % ivb with intense coloration. For stalk imprints, the scale was modified as follows: 1: 0 % < ivb <5 %; 2: 5 % ≤ ivb <30 %; 3: 30 % ≤ ivb <60 %; 4: 60 % ≤ ivb <100 %; and 5:100 % ivb.

### Statistical analyses of phenotypic data

All statistical analyses were conducted using the SAS software (SAS software 9.3, SAS Institute Inc., Cary, NC, USA). Virus incidence (VI) data were transformed by the $ar\sin \left( {\sqrt {\text{VI}} } \right)$ function before statistical analyses to achieve normal distribution of residuals. Independency of cultivar means and variances were controlled for transformed virus incidence data and for virus population density data.

Analyses of variance (ANOVA) were done for each disease resistance trait considering mixed linear models (SAS Mixed procedure) with the restricted maximum likelihood (REML) method. Each trait was analyzed with the two following models considering either both trails together (model 1) or each trial analyzed separately (model 2):Model 1:

$$ {\underline Y}_{{_{ijkl} }} = \mu + {\underline G}_{i} + B_{j(l)} + C_{k(l)} + T_{l} + {\underline {GC}}_{ik(l)} + {\underline {GT}}_{il} + {\underline {\varepsilon}}_{ijkl} $$

Model 2:

$$ {\underline {Y}}_{ijk} = \mu + {\underline {G}}_{i} + B_{j} + C_{k} + {\underline {GC}}_{ik} + {\underline {\varepsilon}}_{ijk} $$

In both models, terms underlined indicate random effects, while other effects are fixed. ${\underline {Y}}_{ijk}$ and ${\underline {Y}}_{ijkl}$ are the phenotype of the *j*th block (*j* = 1, 2, 3) of the *i*th genotype (*i* = 1, 2, …, 189) of *k*th crop cycle (*k* = 1, 3) in each trial or in the *l*th trail (*l* = 1,2); $\mu$ is the overall mean; ${\underline {G}}_{i}$ is the effect of individual *i*; $B_{j(l)}$ and $B_{j}$ are block effect *j* nested or not nested within trial *l*, respectively; $C_{k(l)}$ and $C_{k}$ are crop cycle effect *k* nested or not nested within trial *l*; $T_{l}$ is the trial effect; ${\underline {GC}}_{ik(l)}$ and ${\underline {GC}}_{ik}$ are the genotype x crop cycle interaction nested or not nested within trial *l*; ${\underline {GT}}_{il}$ is the genotype × trial interaction; ${\underline {\varepsilon}}_{ijkl}$ and ${\underline {\varepsilon}}_{ijk}$ are residual errors. In both models individual plot phenotypes within a trial were considered as repeated measurements across crop cycles. The significance of each variance component was tested by a Wald *Z* test using SAS Mixed procedure. The significance of the differences between means of traits per crop cycle within and between trials, were tested with a *t* test under Lsmeans statement in the analysis of model 1 under SAS Mixed procedure.

Broad-sense heritability ($H^{2}$) at an experimental level was evaluated for each trait as the ratio of genotypic variance to phenotypic variance, using the components of variance obtained from the ANOVA as follows:

$$ H^{2} = \sigma_{G}^{2} /(\sigma_{G}^{2} + \sigma_{GC}^{2} /C + \sigma_{GT}^{2} /T + \sigma_{e}^{2} /TCR) \hbox{ for model 1 }$$

$$ H^{2} = \sigma_{G}^{2} /(\sigma_{G}^{2} + \sigma_{GC}^{2} /C + \sigma_{e}^{2} /CR) \hbox{ for model 2 }$$

where $\sigma_{G}^{2}$ = estimation of the genotypic variance; $\sigma_{GC}^{2}$ = estimation of the variance of genotype × crop cycle interaction effect; $\sigma_{GT}^{2}$ = estimation of genotype × trial interaction; $\sigma_{e}^{2}$ = estimation of the residual variance; *C* = number of crop cycles (2); *T* = number of trial (2) and *R* = number of replications (3).

Phenotypic cultivar values within each model were estimated by adding to their empirical best linear unbiased predictors (EBLUPs), the intercept estimate and all fixed effects estimates. Phenotypic values (PhV) were then used to correlate disease resistance traits among and within trials by calculating Pearson correlation coefficients and to perform marker-trait association tests.

### Genotyping data

The Amplified Fragment-Length Polymorphism (AFLP) genotyping was generated using the AFLP^®^ Analysis System I (Invitrogen), with 59 primer pairs, as recommended by the manufacturer with slight modifications described by Hoarau et al. (2001). The Diversity Arrays Technology (DArT) genotyping was produced by the commercial company Diversity Arrays Technology Pty Ltd. (Yarralumla, Australia) using their commercial sugarcane array (Heller-Uszynska et al. 2011). A total of 3,949 polymorphic markers exhibiting frequencies higher than 0.05 and lower than 0.95 (2,400 AFLP + 1,549 DArT) were used for the association mapping tests.

### Marker-trait associations

These tests were conducted using TASSEL (online version 3.0.154) software with genotypic cultivar values either obtained from both trials together (model 1) or from each trail separately (model 2). Two association models were used: the General Linear Model (GLM) with a Q matrix indicative of population structure and the Mixed Linear Model (MLM) with both population structure (Q) and kinship (K) matrix as covariates (Yu et al. 2006). MLM analysis used the compressed mixed linear model approach (Zhang et al. 2010) carried by TASSEL.

Haplotypes were constructed by testing the 3,949 available polymorphic markers in pairwise associations with a bilateral Fisher exact test and by grouping all significant pairwise combinations of markers by transitivity (Raboin et al. 2008). To achieve an overall significance of 5 % using the Bonferroni correction, a nominal significance threshold of *P* = 6.4 × 10^−9^ was applied to each of the 7,795,326 pairwise tests so far performed (3,949 × 3,948/2). We therefore found a total of 463 haplotypes (encompassing 2,480 markers) and 1,469 independent markers.

Q matrix was inferred from DArT markers using the Principal Component Analysis (PCA) approach developed by Patterson et al. (2006) with SAS Princomp procedure. The PCA analyses were performed on the cultivar panel by considering the only 1,509 polymorphic DArT markers showing <10 % of missing data among genotyped cultivars and exhibiting frequencies situated in a (0.05–0.95) interval. These DArT markers represented 619 different haplotypes and independent markers. However, the study of a relatively modest number of markers to cover the large genome of sugarcane (about 120 chromosomes) combined with the rather large linkage disequilibrium (LD) existing among sugarcane cultivars may result in the observation of markers in significant LD that could be separated by large genetic distances (Raboin et al. 2008). In this context, two alternative Q1 and a Q2 matrix were inferred from a PCA analysis either based on the 619 independent DArT haplotype/markers or on all the 1,509 DArT markers, respectively. The effect of these two alternative Q matrixes on GLM model (Q1 versus Q2) and on MLM model (Q1 + K versus Q2 + K) was compared based on the shape of Quantile–Quantile plot of the probabilities of corresponding association tests. The number of PCA axes used to define the Q matrix used in MLM and GLM models was determined according to Zhu and Yu (2009) methodology. Cultivar phenotypes (PhV) were fitted in a linear model with an increasing number of PCA axes and we chose the first most significant axes showing the lowest Bayesian Information Criteria (BIC). In both GLM and MLM models, the Q matrix was considered as a fixed co-variable. In the compressed MLM model, the additional kinship matrix (K) was a Jaccard double similarity matrix computed with Darwin software (Perrier and Jacquemoud-Collet 2006) considering the same 1,509 markers set used for the inference of Q matrix.

Inflation of false-positive associations may occur in genome-wide association studies when the genetic structure is not well modeled. A widely used inflation factor *λ* can be computed by dividing the median test statistic of the quantile–quantile plot by its theoretical median statistic under the null hypothesis of no marker linked to the polymorphism controlling the phenotypes (Price et al. 2010). In our study, the inflation factor *λ* was computed from the Fisher F-statistics provided by association models, following Yu et al. (2006). According to Price et al. (2010) *λ* should be lower than 1.05 to avoid detection of spurious associations. When *λ* ≈ 1, there is no inflation in test statistics.

For both GLM and MLM models with a controlled inflation risk (*λ*), associations were declared significant up to a genome wise type-I error rate (GWER) threshold of *P*_GWER_ = 10 %. For the GLM model, this threshold was defined on the basis of 1,000 permutations. For MLM model, considering the number of independent haplotypes or markers (1,932) this *P*_GWER_ of 10 % corresponded to *P* = (0.10/1,932) using the Bonferroni correction and gives a −log *P* = 4.286.

Finally, in each trial, for each disease resistance trait, we estimated the proportion of the total phenotypic variation that could be explained by the global effect (*R* ^2^) of all the detected markers using a multiple regression analysis (SAS REG procedure) with a stepwise process adding markers one-by-one. Entry and stay option parameters were set up for *F* test marker effect on the regression at *P* = 0.15.

Cumulative markers effect on SCYLV resistance was analyzed by comparing trait means of cultivar classes considering number of markers borne by cultivars, by a *t* test using Lsmeans statement under GLM procedure. Data used were the cultivar PhV means from model 1.

### Search for genes involved in disease resistance

Since sugarcane and sorghum display high micro-colinearity (Jannoo et al. 2007; Wang et al. 2010; Garsmeur et al. 2010), the sequences from selected sugarcane DArT markers were blasted on *Sorghum bicolor* sequence genome using BLASTN 2.2.22 program (Altschul et al. 1997). Genes surrounding sugarcane sequences were searched on sorghum sequence using a window of 100 kb on each side using the Gbrowse function of the web-based tool OryGenesDB available on CIRAD ‘South Green’ Platform (orygenesdb.cirad.fr) which encompasses molecular resources (gene annotation, coding sequence or protein) of several grasses (rice, maize, sorghum). Putative gene function was then searched by identifying homologous genes from *Arabidopsis thaliana* genome using TAIR database (www.arabidopsis.org). Finally, we searched among these *A. thaliana* homologs for genes potentially regulated upon pathogen infection using PathoPlant website (www.pathoplant.de) that hosts microarray expression data involved in plant defence responses (Bülow et al. 2007).

## Results

### Analysis of SCYLV resistance traits in the cultivar panel

SCYLV resistance was assessed in the studied cultivar panel exposed to natural inoculation in the epidemiological context of Guadeloupe. Table 1 summarizes the estimates of the variance components for both ANOVA model 1 (two trials together) and ANOVA model 2 (separate trial analyses). Genetic variances ($\sigma_{G}^{2}$) and residual variances ($\sigma_{e}^{2}$) were always highly significant (*P* < 0.0001) for all resistance traits in both models. Their respective ratio ($\sigma_{G}^{2}$/$\sigma_{e}^{2}$) ranged between modest values for stalk resistance parameters (from 1.10 to 2.20) to higher values for leaf resistance parameters (from 2.53 to 4.40). In model 1, variances of genotype × trial interaction ($\sigma_{GT}^{2}$) and of genotype × crop cycles interactions ($\sigma_{GC}^{2}$) were highly significant (*P* < 0.001 or < 0.0001) for all traits. In the model 2, $\sigma_{GC}^{2}$ was highly significant in trial A for the four resistance traits (at least *P* < 0.001) but failed to be significant in two cases in trial B (stalk SCYLV incidence and density). Broad-sense heritability ($H^{2}$) at the level of both trials (model 1) estimated on an entry-mean basis using three replicates, two crop cycles and two trials ranged between 0.70 and 0.84. Due to the significant variance of interaction of genotype by trial, $H^{2}$ values computed at the experimental level of each trial (model 2) showed a range of 0.83–0.95 that is slightly higher than the former range. Despite small differences between models, all heritability values are high to very high. They indicate that both experimental design and phenotyping protocols provided accurate estimates of the genetic values of the cultivars surveyed and justify testing cultivar estimates produced by both ANOVA models for marker-trait association tests.

**Table 1:** Estimates and significance level of variance components and heritability ($H^{2}$) for SCYLV disease resistance traits

Trait	Model	Trial	Variance components				$H^{2}$
			$\sigma_{G}^{2}$	$\sigma_{GC}^{2}$	$\sigma_{GT}^{2}$	$\sigma_{e}^{2}$	
Leaf SCYLV incidence	1	A and B	0.176****	0.012****	0.049****	0.056****	0.83
	2	A	0.164****	0.017****		0.038****	0.92
	2	B	0.288****	0.008***		0.073****	0.95
Stalk SCYLV incidence	1	A and B	0.112****	0.011***	0.067****	0.102****	0.70
	2	A	0.117****	0.016****		0.093****	0.83
	2	B	0.243****	0.005 NS		0.111****	0.92
Leaf SCYLV density	1	A and B	1.130****	0.122****	0.236****	0.453****	0.84
	2	A	1.251****	0.179****		0.417****	0.89
	2	B	1.492****	0.064***		0.488****	0.93
Stalk SCYLV density	1	A and B	0.469****	0.074****	0.182****	0.380****	0.75
	2	A	0.483****	0.128****		0.354****	0.80
	2	B	0.822****	0.020 NS		0.405****	0.91

***, **** Significant for a Wald *Z* test at *P* = 0.001, *P* = 0.0001 or non-significant (NS) at *P* = 0.05

**Fig. 1:**
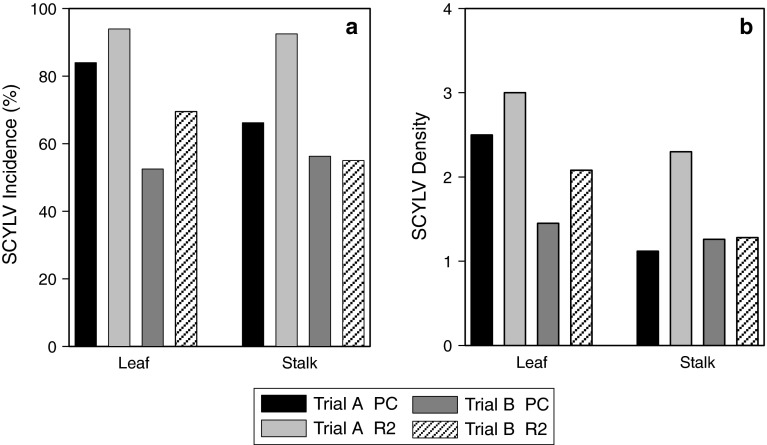
Means of SCYLV resistance traits relative to leaf and stalk samplings in the cultivar panel, in plant cane (PC) and in second ratoon (R2) crop cycles in trials A and B for: virus incidence (**a**) and virus density (**b**)

Cultivar panel means of disease resistance traits in each crop cycle studied for each trial are presented in Fig. 1. When comparing cycle within trials, the mean of foliar disease traits consistently increased upon crop cycles in both trials (*t* test values ranging from −9.6 to −12.4 with *t* = −9.6*; P* < 0.0001). For stalk disease traits, the increase was significant in trial A (*t* values of −16.53 and −25.7 with *P* < 0.0001 for virus incidence and density, respectively) but not in trial B (*t* value of 0.62 and 0.817; *P* = 0.53 and 0.81 for virus incidence and density, respectively). Comparing crop cycle between trials, except for stalk density in plant cane, disease traits were significantly higher in trial A than in trial B for the same cycle with range of *t* value of 2.69 to 15.09 with respective *P* of 0.007–<0.0001.

Correlations between all traits (within and between trials) using cultivar PhV of model2 were highly significant (*P* < 0.0001). Pearson correlation coefficients varied from 0.72 to 0.97 for trait correlations within trials and from 0.53 to 0.75 for trait correlations between trials (Table 2). Relative good correlations between trials were found for SCYLV incidence (*r* = 0.75) and for SCYLV density (*r* = 0.75) in leaf tissues, whereas correlations between trials for SCYLV incidence (*r* = 0.58) and for SCYLV density (*r* = 0.63) in stalk tissues were lower. Correlations of traits within trials, between plant parts (leaf and stalk) were always high: 0.83 and 0.95 for SCYLV incidence in trials A and B, respectively, and 0.76 and 0.90 for SCYLV density in trials A and B, respectively.

**Table 2:** Pearson correlation coefficients between resistant traits using cultivar estimated values

Trial			A				B			
	Trait		SCYLV incidence		SCYLV density		SCYLV incidence		SCYLV density	
		Plant part	Leaf	Stalk	Leaf	Stalk	Leaf	Stalk	Leaf	Stalk
A	SCYLV incidence	Leaf	1	0.83	0.95	0.72				
		Stalk		1	0.81	0.94				
	SCYLV density	Leaf			1	0.76				
		Stalk				1				
B	SCYLV incidence	Leaf	0.75	0.59	0.74	0.53	1	0.95	0.97	0.89
		Stalk	0.71	0.58	0.70	0.55		1	0.92	0.96
	SCYLV density	Leaf	0.71	0.57	0.75	0.55			1	0.90
		Stalk	0.67	0.61	0.68	0.63				1

All correlations are significant at *P* < 0.0001. Correlations within trials (A, B) are above the diagonal and correlations between trials are below the diagonal

### Marker-trait associations

Structure analysis of the cultivar panel with PCA based on the independent 619 DArT haplotypes/markers yielded 11 axes that were significant (*P* < 0.05) with the Tracy–Widom test (T–W). Alternatively PCA with all 1,509 DArT markers yielded 24 significant other axes (T–W test; *P* < 0.05). Fitting each of the 12 estimates of resistance trait (SCYLV incidence and SCYLV density on leaves and stalks in two separate or combined trial analyses) with an increasing number of significant axes showed that minimum BIC was always achieved with only one axe in the first PCA and always three axes in the second PCA. Depending on resistance traits and ANOVA models considered, the first axis of the first PCA explained between 0.6 and 1.3 % of the phenotypic variation and the first three axes of the second PCA explained between 9.8 and 18.6 % of the phenotypic variation. Therefore, the two Q1 and Q2 alternative matrix used as covariate in GLM and MLM models always consisted of the first axis of the first PCA and the first three axes of the second PCA, respectively. Quantile–quantile plots of the 48 different genome-wide association exercises are presented in supplementary material 2.

Inflation factors *λ* of the all associations models are summarized in Table 3. Obviously, whatever the trait, in GLM models test statistics are largely more inflated when using Q1 than when using Q2, since *λ* ranged between 1.395 and 1.848 with Q1 compared to a lower range of 1.051–1.211 with Q2. In all GLM models, *λ* was always above the critical value of 1.05 defined by Price et al. (2010). Therefore, GLM models were not further considered in the presentation of marker-trait association results. As expected, MLM models showed a range of inflation factors (0.951–1.153) lower than in GLM models (1.051–1.848). Models Q2 + K gave lower *λ* values than Q1 + K in a majority of 8 cases over 12. All SCYLV resistance traits together, a total of 10 different MLM models exhibited an efficient control of inflation risk (*λ* < 1.05). Association results further discussed are only based on these 10 MLM models.

**Table 3:** Inflation factors computed for different genome-wide association models assessed on SCYLV resistance traits

Model co-factors	ANOVA		SCYLV incidence		SCYLV density	
	Model	Trial	Leaf	Stalk	Leaf	Stalk
Generalized linear model (GLM)						
Q2	1	A and B	1.211	1.118	1.194	1.051
Q1	1	A and B	1.848	1.708	1.761	1.466
Q2	2	A	1.134	1.088	1.106	1.071
Q1	2	A	1.827	1.557	1.735	1.334
Q2	2	B	1.117	1.103	1.202	1.074
Q1	2	B	1.710	1.541	1.524	1.395
Mixed linear model (MLM)						
Q2 + K	1	A and B	**0.976**	1.069	1.094	**1.011**
Q1 + K	1	A and B	1.153	1.117	1.173	**1.008**
Q2 + K	2	A	1.061	**1.009**	**1.002**	**1.007**
Q1 + K	2	A	**1.037**	**1.000**	1.061	**0.951**
Q2 + K	2	B	1.082	1.073	1.122	**1.020**
Q1 + K	2	B	1.153	1.170	1.223	1.146

Q1- and Q2-matrix are significant axes of PCA analyses inferred either from 619 independent DArT haplotypes/markers or from all 1,509 DArT markers, respectively. They represent a fixed co-factor (Q) for population structure. Co-factor K is the genetic similarity matrix. Inflation factors lower than 1.05 are indicated in bold characters

Table 4 presents the results of these ten GWAS exercises which correspond to different disease parameters, different trial analyses and MLM models. Considering all disease resistance traits and MLM models together, a total of six independent markers were involved in significant associations at a *P*_GWER_ < 0.10. These six independent markers corresponded to three AFLP (aggctc35, agccag14, agccta52) and three DArT markers (B424681, B424690, B424752). Among the six detected markers, only one (B424681) was located on the R570 reference genetic map (homology group VI). The three AFLP markers and B424752 DArT marker were significant in one analysis for a particular trait and MLM model. These four markers showed in one or two alternative trait analyses a −log*P* values higher than 4.00 but below the statistical threshold (4.28). B424681 and B424690 were significant in five and four different trait analyses, respectively. For leaf incidence with the Q1 + K MLM model, these two markers were significant for a −log*P* values of 6.09 and 6.21 which corresponded to *P*_GWER_ values of 0.0015 and 0.0012, respectively.

**Table 4:** Results of marker-trait associations assessed on SCYLV resistance traits for MLM models showing an efficient control of inflation risk (*λ* < 1.05)

Co-factors	ANOVA		Disease parameter		Markers (frequency)					
	Model	Trial	Trait	Plant part	Aggctc35 (0.06)	Agccag14 (0.17)	Agccta52 (0.10)	B424681 (0.20)	B424690 (0.08)	B424752 (0.14)
					−log*P* (*R* ^2^)					
Q2 + K	2	A	Incidence	Stalk	4.05 (0.09)	2.66 (0.05)	3.50 (0.07)	**4.37 (0.09)**	**4.69 (0.10)**	2.84 (0.06)
Q2 + K	2	A	Density	Leaf	2.63 (0.05)	**4.71 (0.10)**	1.95 (0.03)	**4.84 (0.10)**	**4.33 (0.09)**	**5.25 (0.11)**
Q2 + K	2	A	Density	Stalk	3.88 (0.08)	2.66 (0.05)	3.48 (0.07)	3.39 (0.07)	4.01 (0.08)	2.84 (0.06)
Q2 + K	2	B	Density	Stalk	2.54 (0.05)	3.59 (0.07)	2.07 (0.04)	4.15 (0.09)	1.93 (0.03)	1.74 (0.03)
Q2 + K	2	A and B	Incidence	Leaf	3.58 (0.06)	4.01 (0.08)	1.65 (0.02)	2.86 (0.05)	1.58 (0.02)	4.19 (0.08)
Q2 + K	2	A and B	Density	Stalk	3.81 (0.08)	3.72 (0.08)	3.22 (0.06)	**4.47 (0.09)**	3.35 (0.06)	2.67 (0.05)
Q1 + K	2	A	Incidence	Leaf	3.40 (0.07)	3.87 (0.08)	4.00 (0.08)	**6.09 (0.14)**	**6.21 (0.14)**	3.99 (0.08)
Q1 + K	2	A	Incidence	Stalk	**4.47 ** **(0.10)**	2.86 (0.06)	**4.29** **(0.09)**	**4.85** **(0.11)**	**4.65** **(0.10)**	2.82 (0.06)
Q1 + K	2	A	Density	Stalk	3.97 (0.09)	3.01 (0.06)	4.17 (0.09)	3.59 (0.08)	3.78 (0.08)	3.05 (0.06)
Q1 + K	1	A and B	Density	Stalk	3.62 (0.08)	4.00 (0.09)	3.65 (0.08)	3.67 (0.08)	3.10 (0.06)	3.20 (0.06)

In bold character −log(*P*) > 4.28 corresponding to an *P*_GWER_ < 10 % (see material and method section). See legend of Table 3 for the signification of co-factors abbreviations

Frequency of these six markers was relatively low, ranging from 0.06 to 0.20. All alleles showed a favorable effect on SCYLV resistance. According to the modest to high positive correlations between disease resistance traits between and within trials (Table 2), the associations were always in the same direction across all trait analyses, whatever their statistical significance, and even when the percentage of variation (*R* ^2^) explained was almost null.

The proportion of the total phenotypic variation (*R* ^2^) explained individually by significant markers in all trait MLM analyses ranged from 9 to 14 %.

Considering the distribution of the six detected resistant markers within the cultivar panel, number of markers present in individual cultivar ranged from zero to six markers. The number of cultivars bearing from zero to six markers reached 105, 44, 23, 11, 0 and 1 individuals, respectively. Due to their low frequency, cultivars bearing four to six markers were grouped into a single class for further statistic comparisons. As a rule, mean disease incidence values of the five cultivar classes bearing an increasing number of markers (0, 1, 2, 3 and 4–6 markers) decreased gradually: 88, 73, 52, 38 and 16 %, respectively, for leaf incidence; 79, 65, 51, 36 and 24 %, respectively for stalk incidence. A *t* test for each trait showed significant (*P* < 0.5) differences between all classes, except the fourth one that was not significantly different from its two neighbor classes. Moreover, the 35 cultivars bearing multiple markers (from two to six markers) were widely distributed among the 26 different geographical breeding origins represented in the cultivar panel.

The cumulative effects on disease resistance variations of the 6 detected markers were estimated with stepwise multiple regression models (SMRM) using the 176 cultivars with no missing information on detected marker (Table 5). The SMRMs captured between three and five of the six markers depending on trait and trials considered. The percentages of the variation of the panel explained by the markers retained in regression model varied from 20 to 31 %. The most frequently detected marker (B424681) in the GWAS analyses (Table 5) was kept for all disease resistance traits by the SMRM. Interestingly, three markers (aggctc35, agccag14 and B424752) detected only in one MLM trait model (Table 4) were retained in almost all regression models whatever the trait.

**Table 5:** Results of stepwise multiple regressions of markers on SCYLV resistance traits in leaves and stalks, in both trials

Trial	Plant part	Phenotype	*R* ^2^	Aggctc35	Agccag14	Agccta52	B424681	B424690	B424752
A	Leaf	Incidence	0.31	X	X		X	X	X
A	Leaf	Density	0.31	X	X		X	X	X
A	Stalk	Incidence	0.26	X		X	X	X	X
A	Stalk	Density	0.25	X		X	X	X	X
B	Leaf	Incidence	0.22	X	X		X		X
B	Leaf	Density	0.21	X	X		X		X
B	Stalk	Incidence	0.22	X	X		X		X
B	Stalk	Density	0.20	X	X		X		X
A + B	Leaf	Incidence	0.28		X		X		X
A + B	Leaf	Density	0.29	X	X		X		X
A + B	Stalk	Incidence	0.27	X	X		X		X
A + B	Stalk	Density	0.26	X	X		X		X

*R* ^2^ phenotypic variation explained by the model (markers). Markers kept in the stepwise model are indicated by an X

### Search for resistance genes

**Fig. 2:**
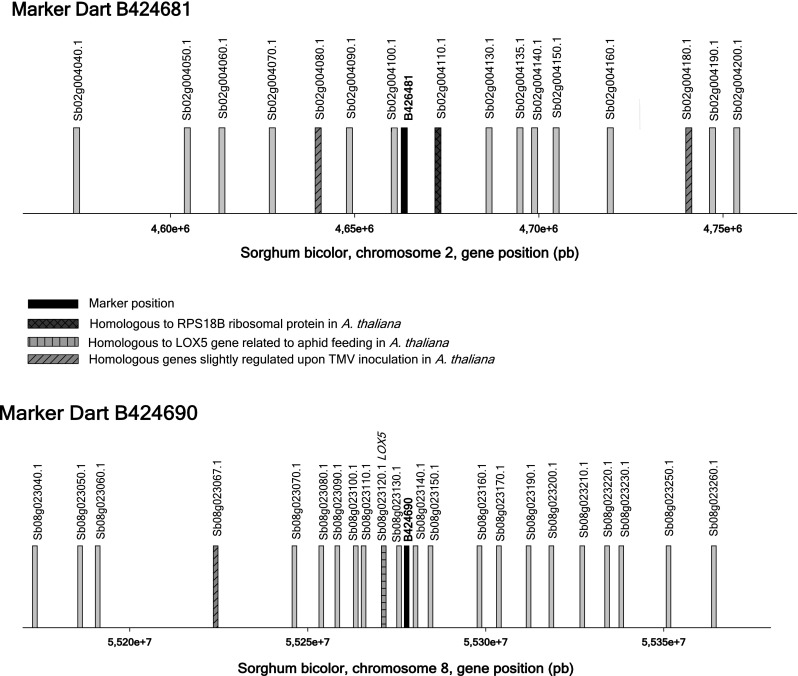
Genes in the sorghum regions (*d* = 100 kb) homologous to two of the sugarcane regions found associated with SCYLV resistance

The sequences of DArT markers B424681, B424690 and B424752 were blasted on the *Sorghum bicolor* genome sequence. Marker B424690 (526 bp) aligns on *S. bicolor* chromosome 8 with convincing BLAST parameters (HSP bit score of 852 and an e-value of 0 with 95 % identity). The B424681 (569 bp) sequence showed 92 % sequence identity with a sequence located on *S. bicolor* chromosome 2 with a HSP bit score of 775 and an e-value of 0. The B424752 sequence of 540 bp, that contains a fragment of a Non-LTR retrotransposon LINE L1, could not be assigned to any specific sorghum chromosome due to partial alignment with multiple sorghum sequences. Figure 2 shows the sets of genes found in *Sorghum* in a region of 100 kb around the sorghum homologs of B424681 and B424690. Very close to B424681 sorghum homolog (at a distant of 11.8 kb), we found a gene homologous to the AT1G34030 gene of *A thaliana* that codes for the 40S ribosomal protein (RPS18B). At a further distance apart from B424681 sorghum homologs (19.9 kb and 78.7 kb), we also recognized two genes whose homologs have unknown functions in *A. thaliana* which are regulated upon the Tobacco mosaic virus (TMV) infection. Another TMV regulated homologous gene with unknown function was found at a distance of 40.7 kb from the B424690 sorghum homolog. Moreover, a gene homologous to a gene coding for a lipoxygenase (LOX5) related to aphid feeding in *A. Thaliana* (AT3G22400) was identified at only 6.4 kb from B424690 sorghum homolog.

## Discussion

To identify genomic regions involved in the resistance of sugarcane to *SCYLV* disease, we studied associations within a panel of 189 modern sugarcane cultivars representative of the worldwide breeding germplasm. Phenotypic assessment of the panel for disease resistance was based on immunological diagnoses of the virus performed on material exposed to natural infections prevailing in the fields in Guadeloupe. To guarantee accurate phenotypic disease rating data in a context of variable natural epidemiological pressures, we invested in two successive repeated field trials phenotyped in two crop cycles using numerous sampling for SCYLV diagnosis. Due to the lack of the literature about the dynamic of the virus within infected sugarcane relative to its circulation and maintenance, we choose to diagnose both leaf and stalk tissues, to grasp a comprehensive picture of the susceptibility level of the cultivar panel and make sure of a proper biological interpretation of the resulting association study.

### Disease resistance assessment relying on natural infection

In both trials, the level of SCYLV contamination was expected to be the result of background infection in cane seeds and further infections caused by SCYLV transmission to plant by the aphid vector. Means of disease trait values were significantly higher in the first trial (trial A) compared to the second trial (trial B) despite the same origin of cane seeds. This difference is attributed to the fact that aphid populations were profuse only in the first crop cycle of trial A, but sparsely observed the other times. Nevertheless, broad-sense heritability values at trial level of all disease resistance traits appeared always high to very high (0.80–0.95) in both trials and also in their combined analysis (0.70–0.84). These findings show efficient control of environmental sources of variations within trial when estimating the genetic values of the cultivars surveyed. A high average value in virus incidence (75 %) was observed in leaves across trials and crop cycles. This level of incidence is similar to incidence values observed in cultivars surveyed in Hawaii (Komor et al. 2010) and Florida (Comstock et al. 1999) that had been exposed for many years to potential virus infection.

### Association tests for SCYLV resistance

Phenotype data from each trial were used to tag for SCYLV resistance alleles through GWAS approaches. In GWAS, population structure has to be correctly modeled to control efficiently the risk of spurious associations (Patterson et al. 2006; Yu et al. 2006). In the polyploid context of sugarcane, the use of dominant markers whose geneticists still depend on, such as the ones we used, constitute an obvious handicap to model accurately population structure. In our study, we attempted a general linear model (GLM) with two alternative population structure co-factors derived from a principle component analysis (PCA) using either all DArT polymorphic markers available or a sampling strategy of independent ones. Both GLM association exercises failed to control an inflation risk of test statistics and possible spurious associations. On the contrary, mixed linear models (MLM) with the addition of a kinship matrix covariate, allowed a strict control of the inflation of test statistic (*λ* < 1.05) on ten occasions.

These ten MLM genome-wide association studies revealed six independent significant markers linked to one or several SCYLV resistance parameters at a genome-wide error rate (GWER) lower than 10 %. Among them two DArT markers (B424681 and B424690) were detected at a *P*_GWER_ of 0.0015 and 0.0012 for a particular resistance trait (disease leaf incidence). All the six markers were associated with resistance. These six markers were present in the cultivar panel at a low frequency (6–20 %). The low frequency of resistance alleles in our worldwide cultivar panel combined with the high virus incidence mean reflects (1) the absence of selection in breeding programs due to the recent spread of the disease (Komor et al. 2010) and (2) a probable relative scarcity of sources of resistance available in the modern interspecific sugarcane germplasm. Individual allele effects associated with yellow leaf resistance traits explained from 9 to 14 % of the total variation of the cultivar panel. These effect sizes which are overestimated regarding the modest panel size of the panel were similar to those obtained in previous sugarcane association study of Wei et al. (2006) relative to three sugarcane disease resistance (Fiji disease, leaf-scald and pachymetra root rot) and based on a small panel (154 accessions).

Multiple regression analysis showed that the part of variation in yellow leaf resistance traits, measured by leaf diagnosis, varied from 20 to 31 % depending on trait assessed. Our results are similar to Wei et al. (2006) association study which detected in sugarcane four markers relative to the resistance of another vectored virus disease (Fiji disease) explaining together 32 % of the variation in the cultivar panel surveyed. The large part of the variation yet left unexplained might be attributed to the addition of several reasons: (1) a certain number of perceptible alleles of interest might have been missed due to an insufficient marker density; (2) QTL of medium effect in a single variety might exhibit a too small quantitative effect in the context of the association study to be detected whatever marker density; (3) rare alleles of significant effects in a few cultivar might have also been missed and fell in the “missing heritability” category (Yang et al. 2010); (4) finally, depending on the narrow-sense heritability of the disease traits, an undetermined and inaccessible proportion of the variance might be due to more or less complex allele interactions.

The modest part of variation explained in our study could be explained by a lack of power in our association tests. Four main factors limited the detection of markers: (1) the modest size of the panel: it allowed tagging a few alleles that are believed to be some of the most important ones. A larger panel should substantially improve the statistical power of detection of medium- and small-marker effects (Long and Langley 1999). (2) The confounding effect of polyploidy: in polyploid crop species, detection of linkage disequilibrium (LD) between a QTL and a marker in its vicinity could be missed due to unbalanced frequencies between QTL and marker. The risk of undetected genuine linkage is expected to increase with polyploidy level. In the context of the high ploidy level of sugarcane, such undetected LD was shown to be common with dominant markers (Fig. 3 in Raboin et al. 2008). (3) The use of dominant markers: their dichotomous nature (presence versus absence) involves a loss of power in QTL detection compared to co-dominant markers which enable homozygotes and heterozygotes to be distinguished. In out-crossing polyploidy species such as sugarcane, only markers segregating in very low dosage (simplex or duplex) behind the dominant ‘presence’ phenotypes are believed to be sufficiently informative to tag alleles of agronomic interest. The fact that the six markers found in our study were all in low frequency in the panel (0.06–0.20) gave credit to this intuitive believe. (4) Finally, the number of markers we used, despite substantial, was probably far from sufficient to tag all the haplotype diversity that segregate in our panel. Capturing the polymorphic fraction of the genome is especially challenging in the polyploid species, where copies of homologous chromosomes “dilute” the polymorphism.

These well-known constraints in the genetic analysis of polyploids that severely limit the power of QTL detection could be overcome with the advent of single-nucleotide polymorphism (SNP) technology and statistical innovations in data interpretation dedicated to polyploids. Recently, Hackett et al. (2013) adapted current methodologies of linkage and QTL analyses of diploid to autotetraploid potato using SNP dosage. Interestingly, Serang et al. (2012) developed a formal statistical method allowing SNP genotype calling and allele dosage estimates in polyploids. A Bayesian model based on comparative allele signal intensities, gives access to genotype configuration with any bi-allelic SNP loci of any sort of polyploids even of the complex sugarcane (Garcia et al. 2013). Moving from dominant markers to bi-allelic SNP with dosage information should improve the power of association studies due to: (1) the inference of more realistic genetic structure likely reducing the inflation of test statistics, (2) the opportunity to conceive refined genetic model to analyze within loci effects.

### Candidate genes in the vicinity of DArT markers

Resistance of plant to a vectored virus might result from antixenosis or antibiosis phenomenon as observed in sugarcane against *M. sacchari* (Fartek et al. 2012) or from the limitation of the multiplication and the movement of the virus within the host (Kang et al. 2005). We had the opportunity to search for genes in the vicinity of two significant DArT markers that blasted on a unique locus in the genome of sorghum. In the sorghum regions homolog to the sugarcane regions associated with SCYLV resistance, we discover a few genes involved in Arabidopsis aphid or virus interaction.

One of these genes encodes for a particular lipoxygenase (LOX5) favoring aphid feeding and colonization of Arabidopsis foliage by a peach aphid species (Nalam et al. 2012). Lipoxygenase (LOXs) multi gene family catalyze hydroperoxidation of free fatty acids into diverse biologically actives compounds named oxylipins. Interestingly, some isoforms of LOX genes are known to be involved in defence response to pathogens by producing signaling compounds and antimicrobial compounds (Liavonchanka and Feussner 2006). Moreover, a LOX isoform recently characterized in tea plant was shown to be involved in plant response following tea feeding by phloem-feeder pests among which an aphis species (Liu and Han 2010).

Another candidate gene that we detected is a homolog of *A. thaliana* gene encoding a 40S ribosomal protein S18 (RPS18B). Interestingly the 40S ribosomal subunit is involved in the initiation of the translation of viral RNA. Virus multiplication depends on the translation machinery of its host and interferences with numerous specific mechanism generated by the virus that could be part of the host-range control of plant viruses (Fütterer and Hohn 1996; Thiebeauld et al. 2007). The hypothesis that this corresponding gene is implicated in virus population regulation is congruent with the high frequency of the detection of the corresponding marker (B424681) by GWAS and multiple regression analysis.

Finally, among the candidate genes detected we found three genes, homologous to genes with unknown functions that are regulated upon the Tobacco mosaic virus (TMV) infection in *A. thaliana*.

### Prospects about breeding applications

Previously, the unique genetic study carried out to characterize yellow leaf resistance in sugarcane was on the basis of a biparental cross between a susceptible and a resistant cultivar (Costet et al. 2012). A major quantitative resistance allele (QRA) to SCYLV, named *Ryl*1, was tagged in the resistant parent (MQ76-53) thanks to three AFLP markers. This resistant clone represents an atypical semi-exotic sugarcane genotype since it is derived from a cross between the old domesticated sugar-producing line ‘Trojan’ (CO 270 × *S. officinarum*) and the wild accession (*S. spontaneum*) SES 528 (Raboin et al. 2006). MQ76-53 was present in our association panel, but none of the six resistant markers we detected was detected in MQ76-53 genome. As mentioned by Costet et al. (2012), the most significant markers associated with *Ryl*1 in MQ76-53, were absent in the small cultivar panel (*n* = 72) studied by Raboin et al. (2008), that was representative of the worldwide current sugar-producing breeding germplasm. There hence is a high chance that *Ryl*1 is present at a very low frequency in our current panel that is also representative of the worldwide sugar-producing germplasm used in breeding, and, the probability of the detection of this major resistance gene is believed to be unlikely.

Among the six markers detected, two markers (B424681 and B424690) were repeatedly found significant (*P*_GWER_ < 0.01) up to highly significant (*P*_GWER_ < 0.0015). Regarding their effect size, these loci have a high chance to represent major resistance alleles in most of the cultivars that bear them. Some of markers found in this study might also tag genuine alleles of resistance with significant quantitative effect in a single cultivar genome. It would be interesting to test the significance of these candidate markers in ad hoc QTL studies between polymorphic parents for each marker to check the existence of related major alleles and evaluate their effect size. Identifying new accessions bearing additional major resistant QTLs different from *Ryl*1 in germplasm less exotic than MQ76-53, would allow for greater flexibility in breeding programs as well as allowing sustainable pyramid strategies with different resistance sources.

## Electronic supplementary material

Below is the link to the electronic supplementary material.

Supplementary material 1 (DOCX 19 kb)

Supplementary material 2 (DOCX 650 kb)
