# Supplementary material for: Prospecting sugarcane resistance to Sugarcane yellow leaf virus by genome-wide association
Source: Theor Appl Genet. 2014 Jun 12;127(8):1719–32. doi: 10.1007/s00122-014-2334-7 (PMC4110414; doi:10.1007/s00122-014-2334-7)
Supplement: Supplementary file 1 — Supplementary material 1 (DOCX 19 kb) [file 122_2014_2334_MOESM1_ESM.docx]

**Supplementary material 1**

**Suppl. Table**: Sugarcane cultivars used in GWAS. Name of cultivar and origin (country of breeding program)

| **Cultivar** | **Origin** | **Cultivar** | **Origin** | **Cultivar** | **Origin** | **Cultivar** | **Origin** |
| --- | --- | --- | --- | --- | --- | --- | --- |
| B41227 | Barbados | BJ82118 | Barbados-Jamaica | FR90253 | France (Guadeloupe) | PHIL66-07 | Philippines |
| B53267 | Barbados | BJ8510 | Barbados-Jamaica | FR90771 | France (Guadeloupe) | POJ213 | Java |
| B54001 | Barbados | BR8177 | Barbados-Dom. Rep. | FR91253 | France (Guadeloupe) | PR1059 | Puerto-Rico |
| B54215 | Barbados | BT902495 | Barbados-Trinidad | FR91576 | France (Guadeloupe) | PR1116 | Puerto-Rico |
| B60182 | Barbados | CB47-89 | Brazil | FR91699 | France (Guadeloupe) | PS56 | Java |
| B60267 | Barbados | CL41-223 | USA | FR9422 | France (Guadeloupe) | PS58 | Java |
| B62163 | Barbados | Co281 | India | FR94119 | France (Guadeloupe) | PS60 | Java |
| B64136 | Barbados | Co290 | India | FR941006 | France (Guadeloupe) | PS61 | Java |
| B64277 | Barbados | Co312 | India | FR9525 | France (Guadeloupe) | Q100 | Australia |
| B6623 | Barbados | Co740 | India | H32-8560 | USA (Hawaii) | Q102 | Australia |
| B70466 | Barbados | Co775 | India | H68-1158 | USA (Hawaii) | Q108 | Australia |
| B70533 | Barbados | Co842 | India | H69-9092 | USA (Hawaii) | Q112 | Australia |
| B70571 | Barbados | Co997 | India | IAC64-257 | Brazil | Q119 | Australia |
| B711001 | Barbados | Co1148 | India | JA6420 | Cuba | Q122 | Australia |
| B7410 | Barbados | Co1186 | India | L6697 | Pakistan | Q127 | Australia |
| B7460 | Barbados | Co1223 | India | LF52-3082 | Fiji | Q128 | Australia |
| B7678 | Barbados | Co62175 | India | LF53-4789 | Fiji | Q134 | Australia |
| B76359 | Barbados | Co6415 | India | LF56-26 | Fiji | Q138 | Australia |
| B77195 | Barbados | Co6806 | India | LF56-51 | Fiji | Q140 | Australia |
| B78124 | Barbados | CP52-43 | USA | LF61-00 | Fiji | Q175 | Australia |
| B78527 | Barbados | CP57-614* | USA | LF61-05 | Fiji | R331 | France (Reunion) |
| B7945 | Barbados | CP59-73 | USA | LF63-33 | Fiji | R366 | France (Reunion) |
| B79136 | Barbados | CP61-84 | USA | LF65-3611 | Fiji | R526 | France (Reunion) |
| B82126 | Barbados | CP62-258 | USA | LF65-3661 | Fiji | R570 | France (Reunion) |
| B85356 | Barbados | CP67-413 | USA | M31/45* | Mauritius | R572 | France (Reunion) |
| B85747 | Barbados | CP69-1062 | USA | M409/51 | Mauritius | R573 | France (Reunion) |
| B85764 | Barbados | CP72-355 | USA | M13/56 | Mauritius | R575 | France (Reunion) |
| B85902 | Barbados | CP76-331 | USA | M377/56 | Mauritius | R576 | France (Reunion) |
| B8649 | Barbados | CP84-1198 | USA | M907/61 | Mauritius | R577 | France (Reunion) |
| B86185 | Barbados | CP85-1432 | USA | M1227/62 | Mauritius | R579 | France (Reunion) |
| B86517 | Barbados | CP86-1633 | USA | M695/69 | Mauritius | R831592 | France (Reunion) |
| B86699 | Barbados | D8804 | Guyana | M292/70 | Mauritius | R84693 | France (Reunion) |
| B8701 | Barbados | D8882 | Guyana | MEX68-200 | Mexico | R841269 | France (Reunion) |
| B871296 | Barbados | DB5505 | Guyana-Barbados | MEX71-1235 | Mexico | ROC1 | China |
| B8930 | Barbados | DB60377 | Guyana-Barbados | MEX73-523 | Mexico | ROC3 | China |
| B90246 | Barbados | DB63237 | Guyana-Barbados | MQ76/53 | Australia | ROC6 | China |
| B93440 | Barbados | DB7047 | Guyana-Barbados | MY51-05 | Cuba | ROC7 | China |
| B93873 | Barbados | DB74208 | Guyana-Barbados | MY54-129 | Cuba | ROC9 | China |
| BBZ80219 | Barbados-Belize | DB8286 | Guyana-Barbados | N11 | South Africa | S17 | Saipan |
| BBZ8108 | Barbados-Belize | DB82113 | Guyana-Barbados | N14 | South Africa | SP71-8210 | Brazil |
| BBZ85102 | Barbados-Belize | DB86124 | Guyana-Barbados | N15 | South Africa | SP73-3108 | Brazil |
| BJ6902 | Barbados-Jamaica | F148 | Taiwan | N17 | South Africa | SP78-3137 | Brazil |
| BJ7003 | Barbados-Jamaica | F156 | Taiwan | N19 | South Africa | SP79-1011* | Brazil |
| BJ7013 | Barbados-Jamaica | F160 | Taiwan | N22 | South Africa | SP80-1842 | Brazil |
| BJ7938 | Barbados-Jamaica | F175 | Taiwan | N52-219 | South Africa | TUC74-06 | Argentina |
| BJ8226 | Barbados-Jamaica | FR8903 | France (Guadeloupe) | N53-216 | South Africa | WI8934 | Barbados |
| BJ8231 | Barbados-Jamaica | FR9027 | France (Guadeloupe) | NCo334 | India |  |  |
| BJ8248 | Barbados-Jamaica | FR90219 | France (Guadeloupe) |  |  |  |  |

*Not use in the GWAS exercises due to insufficient genotyping data
