# Supplementary material for: Prospecting sugarcane resistance to Sugarcane yellow leaf virus by genome-wide association
Source: Theor Appl Genet. 2014 Jun 12;127(8):1719–32. doi: 10.1007/s00122-014-2334-7 (PMC4110414; doi:10.1007/s00122-014-2334-7)
Supplement: Supplementary file 2 — Supplementary material 2 (DOCX 650 kb) [file 122_2014_2334_MOESM2_ESM.docx]

**Supplementary material 2**

**Supplementary material 2 continued**

**Supplementary figure**  : Quantile-Quantile plots of probabilities comparing six models of genome-wide association mapping applied on four different SCYLV resistance traits and using two alternative fixed co-factor Q for population structure : Q1 versus Q2 using the significant axes of a PCA based either on 619 independent DArT haplotype/markers or on all 1509 DArT markers, respectively. GLM consist in linear models using i) Q1-matrix or Q2-matrix with cultivar estimates of trials A and B ii) Q1-matrix or Q2-matrix with cultivar estimates of either trail A or trial B. MLM consist in mixed linear models using alternatively the two fixed co-factor Q combined with a similarity matrix K specified as model co-variance matrix.
